# Exploring the inhibitory potential of *in silico*-designed small peptides on *Helicobacter pylori* Hp0231 (DsbK), a periplasmic oxidoreductase involved in disulfide bond formation

**DOI:** 10.3389/fmolb.2023.1335704

**Published:** 2024-01-11

**Authors:** Paula Roszczenko-Jasińska, Artur Giełdoń, Dominika Mazur, Marta Spodzieja, Maciej Plichta, Cezary Czaplewski, Wojciech Bal, Elzbieta K. Jagusztyn-Krynicka, Dariusz Bartosik

**Affiliations:** ^1^ Department of Bacterial Genetics, Institute of Microbiology, Faculty of Biology, University of Warsaw, Warsaw, Poland; ^2^ Faculty of Chemistry, University of Gdańsk, Gdańsk, Poland; ^3^ Institute of Biochemistry and Biophysics, Polish Academy of Sciences, Warsaw, Poland

**Keywords:** *Helicobacter pylori*, disulfide bond proteins, Hp0231 oxidoreductase, peptide-based inhibitors, Dsb targeting inhibitors, structure-based drug design

## Abstract

**Introduction:** *Helicobacter pylori* is a bacterium that colonizes the gastric epithelium, which affects millions of people worldwide. *H. pylori* infection can lead to various gastrointestinal diseases, including gastric adenocarcinoma and mucosa-associated lymphoid tissue lymphoma. Conventional antibiotic therapies face challenges due to increasing antibiotic resistance and patient non-compliance, necessitating the exploration of alternative treatment approaches. In this study, we focused on Hp0231 (DsbK), an essential component of the *H. pylori* Dsb (disulfide bond) oxidative pathway, and investigated peptide-based inhibition as a potential therapeutic strategy.

**Methods:** Three inhibitory peptides designed by computational modeling were evaluated for their effectiveness using a time-resolved fluorescence assay. We also examined the binding affinity between Hp0231 and the peptides using microscale thermophoresis.

**Results and discussion:** Our findings demonstrate that *in silico*-designed synthetic peptides can effectively inhibit Hp0231-mediated peptide oxidation. Targeting Hp0231 oxidase activity could attenuate *H. pylori* virulence without compromising bacterial viability. Therefore, peptide-based inhibitors of Hp0231 could be candidates for the development of new targeted strategy, which does not influence the composition of the natural human microbiome, but deprive the bacterium of its pathogenic properties.

## 1 Introduction

*Helicobacter pylori* (phylum *Campylobacterota*, class *Epsilonproteobacteria*) is a Gram-negative helical-shaped bacterium capable of colonizing the gastric epithelium of humans. While the prevalence of *H. pylori* infection varies in different regions of the world, it affects approximately half of the human population, which raises the question of whether this bacterium should be regarded as a colonizer or a pathogen (Blaser, 2008; Cover and Blaser, 2009). Colonization of the gastric mucosa by *H. pylori* can result in a variety of upper gastrointestinal tract disorders, although only a subset of infected individuals develop disease. Infection causes inflammatory reactions in the gastric mucosa, which leads to peptic ulcers in about 10% of cases (De Falco et al., 2015).

In 1994 the International Agency for Research on Cancer (IARC), a subordinate organization of the World Health Organization (WHO), classified *H. pylori* as a class I carcinogen (IARC, 1994). Approximately 1% and 0.1% of *H. pylori* infections are associated with the development of gastric adenocarcinoma and mucosa-associated lymphoid tissue lymphoma, respectively (De Falco et al., 2015). The severity of *H. pylori*-associated disease appears to be highly dependent on the bacterial genotype (Cover, 2016). Other important factors that influence infection include the health status and genotype of the host, and environmental conditions such as nutrition (Noto et al., 2013).

International guidelines recommend different drug regimens to treat *H. pylori* infections (Ho et al., 2022). However, due to varying levels of antibiotic resistance in different regions of the world, the therapy should be tailored to local resistance data, particularly clarithromycin resistance rates. Recommended treatments include triple, quadruple or sequential therapy regimens that usually involve the use of at least two different antibiotics and proton pump inhibitors (Roszczenko-Jasińska et al., 2020). Despite these interventions, *H. pylori* eradication fails in more than 20% of patients, and this high failure rate can be attributed to the resistance of this pathogen to different antibiotics, including clarithromycin, metronidazole, amoxicillin and levofloxacin, which correlates with overall antibiotic consumption in the human population. Lack of patient compliance can also impede effective therapy.

The WHO has recognized clarithromycin-resistant strains of *H. pylori* as one of twelve priority pathogens for which novel antibiotics or antibacterial therapies are urgently required (World Health Organization, 2017). Global screening approaches combining genomic, proteomic and metabolic analyses of multiple *H. pylori* strains, have identified several potential new drug targets that may help to eliminate *H. pylori* without interfering with the physiological microbiota (Sarkar et al., 2012; Nammi et al., 2016; Nammi et al., 2017; Uddin and Khalil, 2020). An alternative approach involves targeting virulence factors rather than molecules crucial for bacterial viability. Novel therapeutic agents that specifically target bacterial virulence could have the additional benefit of reducing the development of antibiotic resistance (Rasko and Sperandio, 2010). In terms of their mode of action, these agents can be classified into two categories: those that target a specific virulence factor and those that target broader bacterial processes implicated in pathogen virulence, such as the two-component systems, secretion systems or post-translational protein modifications.

Dsb (disulfide bond) proteins, particularly those involved in the oxidative pathways, have emerged as potential targets for the development of anti-virulence drugs. These proteins catalyze the formation of disulfide bonds between cysteine residues within a polypeptide chain, which plays a key role in determining the structure and activity of the substrate protein. In many Gram-negative bacteria Dsb proteins play a pivotal role in ensuring the proper assembly and folding of various virulence factors, such as toxins, adhesins, flagella and components of secretion systems. It has been demonstrated that the inactivation of *dsb* genes attenuates many pathogens (Heras et al., 2009; Smith et al., 2016; Bocian-Ostrzycka et al., 2017). Therefore, blocking the interactions of Dsb proteins with their polypeptide substrates or redox partners may inhibit the formation of virulence determinants. Since Dsb proteins are located in the periplasm, they are readily accessible to small molecule inhibitors.

Periplasmic proteins are convenient targets for the development of new antivirulence agents or antibiotic adjuvants against Gram-negative bacteria (Pandeya et al., 2020). The recently exploited periplasmic targets include, e.g., AcrA—acriflavine resistance protein A (its inactivation results in increased sensitivity to many antimicrobials) (Russel Lewis et al., 2023), MtrD—resistance-nodulation-cell division (RND) family transporter protein (its inhibition impairs the function of the multidrug efflux pomp) (Lyu et al., 2022), SurA—periplasmic chaperone (its inhibition results in increased antibiotic susceptibility and decreased virulence) (Bell et al., 2018), LptA—lipopolysaccharide transport protein (its inhibition alters or blocs transport of lipopolysaccharides) (Sinha et al., 2022), and PelA—deacylase (its inhibition leads to disruption of exopolysaccharide production and biofilm formation) (Razvi et al., 2023).

The Dsb system of *H. pylori* differs from those of other Gram-negative bacteria. It lacks the conventional monomeric oxidoreductase DsbA and its redox partner DsbB (both involved in the Dsb oxidative pathway) as well as typical DsbC and DsbD proteins, involved in the isomerization pathway. *H. pylori* encodes only two periplasmic proteins with the thioredoxin fold (common in Dsb enzymes), namely, Hp0377 and Hp0231 (DsbK), and the membrane-located DsbB-like protein encoded by the *hp0595* gene. Hp0377 belongs to the CcmG (cytochrome c maturation) protein family (Grzeszczuk et al., 2018). However, unlike other members of this family, it plays a dual role—both in apocytochrome c reduction and disulfide isomerization. Hp0377 exists in both monomeric and dimeric forms, and is maintained in its reduced state by CcdA (Hp0265)—an integral membrane protein with DsbD properties (Roszczenko et al., 2015).

For several years our research has focused on the Dsb pathways of *H. pylori* (e.g., Roszczenko et al., 2012; Roszczenko et al., 2015; Bocian-Ostrzycka et al., 2015; Bocian-Ostrzycka et al., 2017; Grzeszczuk et al., 2018; Roszczenko-Jasińska et al., 2020). In a previous study we showed that Hp0231 (DsbK) is the main oxidoreductase involved in the Dsb oxidizing pathway (Roszczenko et al., 2012). The structure of this dimeric protein (Yoon et al., 2011) revealed similarity to the V-shaped proteins, including EcDsbC and EcDsbG of *Escherichia coli*. We demonstrated that the Hp0231 dimerization domain ensures contact of the oxidoreductase with its substrates, since a truncated form lacking this domain was unable to complement the *H. pylori hp0231* mutant, but it could act in an EcDsbB-dependent manner in *E. coli* (Bocian-Ostrzycka et al., 2015).

The activity of *H. pylori* Dsb proteins is crucial in forming the correct conformation of multiple outer membrane proteins (OMPs) that play an important role in bacterial adhesion (Pang et al., 2014; Javaheri et al., 2016; Moonens et al., 2016). For example, the OMP HopQ, through its binding to human CECAM receptors (frequent targets of infection-causing bacteria), facilitates the adhesion of this bacterium to epithelial cells (Grzeszczuk et al., 2018). Inactivation of the *hp0231* gene also results in (i) impaired translocation of the *H. pylori* CagA protein (cytotoxin-associated protein A), which is associated with gastric carcinoma in the host, (ii) the loss of *H. pylori*-induced production of interleukin 8 (IL-8) (one of the major mediators of the inflammatory response), and (iii) increased sensitivity of *H. pylori* cells to oxidative stress (Lester et al., 2015). Moreover, an *hp0231*-deficient strain of *H. pylori* was unable to colonize the gastric mucosa of mice (Zhong et al., 2016). The above data strongly suggest that targeting oxidoreductase Hp0231 may reduce or block the pathogenic properties of *H. pylori*.

The aim of this study was to identify peptide-based inhibitors that block Hp0231 oxidase activity. To achieve this we (i) designed potential inhibitors using a grain coarse modeling approach, (ii) evaluated their inhibitory potential in a time-resolved fluorescence assay, and (iii) tested their binding affinity with the Hp0231 oxidoreductase to assess the specificity of the interactions. The promising results obtained represent the first step in the development of new therapeutic strategies to combat *H. pylori* infections.

## 2 Materials and methods

### 2.1 Bacterial strains, plasmids, media and growth conditions

The bacterial strains used in this study are listed in Table 1. *Escherichia coli* TG1 was used as a host for the construction of plasmid pUWM2200 (pET28a expression vector with *hp0231*_*APHA*_ gene, encoding Hp0231_APHA_—inactive form of the Hp0231 protein). The *E*. *coli* strain Rosetta (DE3)pLacI was used as the host for plasmids pUWM2200 and pUWM525 (pET28a expression vector with *hp0231* gene, encoding Hp0231 protein) (Roszczenko et al., 2012) in the recombinant protein expression experiments. All strains were grown at 37°C on lysogeny broth (LB) medium or ZY auto-induction medium (Studier, 2005). As required, growth media were supplemented with antibiotics at the following concentrations: 30 μg/mL kanamycin and 20 μg/mL chloramphenicol.

**TABLE 1:** Bacterial strains used in this study.

Name	Relevant characteristics	Source
***E. coli* TG1**	*supE44 hsd*Δ *5 thi* Δ(*lac* ^*-*^ *proAB*) F’ [*traD36 proAB* ^+^ *lacI* ^*q*^ *lacZΔM15*]	Sambrook and Russell (2001)
***E. coli* Rosetta (DE3)pLacI**	F^−^ *ompT hsdSB* (rB^−^ mB^−^) *gal dcm* pRARE (Cm^r^)	Novagen
***E. coli* PR525**	Rosetta (DE3)pLacI carrying pUWM525 (*hp0231* in pET28a)	Roszczenko et al. (2012)
***E. coli* PR2200**	Rosetta (DE3)pLacI carrying pUWM2200 (*hp0231*_*APHA*_ in pET28a)	This study

### 2.2 General DNA manipulation

Standard DNA manipulations were carried out as described in Sambrook and Russell (2001) or according to instructions supplied with kits or reagents. Polymerase chain reactions (PCRs) were performed with PrimeStar HS DNA Polymerase (Takara) under standard conditions. Synthesis of synthetic oligonucleotides and DNA sequencing were performed by Genomed S.A. (Warsaw, Poland). To obtain protein Hp0231_APHA_, a derivative of Hp0231 mutated in the CPHC catalytic motif, recombinant expression plasmid pUWM2200 was constructed. This was created by site-directed mutagenesis of pUWM525 (Roszczenko, et al., 2012), created by cloning the *hp0231* gene (without its native promoter and 5′ region encoding the signal sequence) in expression vector pET-28a (Novagen). Mutations to replace both cysteine residues in the catalytic motif with alanine (CPHC → APHA) were generated with the Quick Change Site-Directed Mutagenesis Kit (Stratagene), using 100 ng of pUWM525 and 125 ng of each oligonucleotide primer: AXXA_231F (5′-CTT​TAT​ATT​GTC​TCT​GAT​CCC​ATG​GCC​CCA​CAT​GC CCA​AAA​AGA​GCT​CAC​TAA​ACT​TAG-3′) and AXXA_231R (5′-CTA​AGT​TTA​GTG​AGC​TCT​TT TTG​GGC​ATG​TGG​GGC​CAT​GGG​ATC​AGA​GAC​AAT​ATA​AAG-3′). The mutation was verified by DNA sequencing.

### 2.3 Protein and peptide analyses and biochemical assays

#### 2.3.1 Expression and purification of recombinant Hp0231 and Hp0231_APHA_

Hp0231 and Hp0231_APHA_ proteins carrying C-terminal 6xHis tags were overexpressed in *E. coli* Rosetta (DE3)pLacI-derived strains PR525 and PR2200 (harboring pUWM525 and pUWM2200, respectively) following auto-induction (Studier, 2005). The starter cultures (10 mL) were propagated for 5 h with shaking in auto-induction medium ZY (supplemented with kanamycin—plasmid selection antibiotic). The culture were then diluted (at a ratio of 1:100) in 400 mL of ZY medium (supplemented with 0.5% glycerol, 0.05% glucose and 0.2% lactose) and cultured overnight (16–18 h) with shaking at 37°C. After induction, cultures were centrifuged (4,000 *g*) and the cell pellets were resuspended in 50 mM sodium phosphate (pH 8.0), 300 mM NaCl, 10 mM imidazole. The cells were then disrupted by ultrasonication. The cell lysates were centrifuged (8,000 *g*) and the resulting supernatants were applied onto Bio-Scale Mini Profinity IMAC^®^ Cartridges (Bio-Rad) containing Ni-charged resin. The proteins were eluted with an imidazole gradient, using the NGC chromatography system (Bio-Rad). For further purification the proteins were loaded onto ENrich®SEC 70 size exclusion columns (Bio-Rad) and eluted with a buffer composed of 20 mM HEPES and 150 mM NaCl (pH 7.4).

#### 2.3.2 Molecular dynamics simulations of inhibitory peptide interactions with Hp0231 protein using a coarse-grained UNRES model

To study interactions between inhibitory peptides and protein Hp0231, molecular dynamics simulations were conducted with the physics-based coarse-grained UNRES model (Liwo et al., 2014). The scale-consistent NEWCT-9 version of the UNRES force field was used (Liwo et al., 2019). A coarse-grained model was selected due to its ability to simulate conformational changes faster than the all-atom model. In the initial structure, the extended chain of the peptide was located close to the catalytic site within the crystal structure of the Hp0231 protein (PDB code 3TDG). Log-Gaussian restraints were imposed on the protein structure. The duration of the simulation was 2 ns. The temperature of 300 K was controlled by the Langevin thermostat. The convergence of the interaction energy between the inhibitory peptide and Hp0231 protein was monitored during simulations. The selected complexes with the strongest interactions between peptide and protein were converted to the all-atom structures using the PULCHRA (Rotkiewicz and Skolnick, 2008) and SCWRL (Wang et al., 2008) algorithms, and refined by running the minimization with the ff14SB AMBER force field and Generalized Born Surface Area (GBSA) implicit-solvent model.

#### 2.3.3 Synthesis of inhibitory peptides

Peptides (WAW6 Ac-HQSALYEL-NH_2_, WAW7 Ac-HQSCAYEL-NH_2_, WAW8 Ac-HQSCACEL-NH_2_) were synthesized using the solid phase peptide synthesis (SPPS) procedure, performed with an automated microwave peptide synthesizer (CEM, Liberty Blue) and the Fmoc/tBu strategy. Rink Amide ProTide Resin (LL) (1.25 g with a capacity of 0.21 mmol/g; CEM, Liberty Blue) was used as the support. During the synthesis, each of the amino acids was coupled twice, using a 5-fold excess relative to the amount of resin deposition. The following reagents were used: 0.5 M DIC in DMF and 1.0 M OxymaPure as coupling reagents, 20% piperidine in DMF for Fmoc-deprotection, and DMF to wash the resin between the deprotection and coupling steps. After synthesis, the peptide N-terminal amino group was acetylated using N-acetylimidazole in DMF (1.6 g/1.25 g of resin) at room temperature for 24 h. Next, the peptidyl resin was washed using DMF and methanol. The peptides were cleaved from the resin by continuous shaking in a mixture of 88% TFA, 5% H_2_O, 5% phenol, and 2% TIPSI (20 mL/1.25 g of resin) at room temperature for 3 h. Then the peptides were separated from the resin by filtration and precipitated using Et_2_O. Following centrifugation at 2608 × g for 15 min at 4°C, the Et_2_O phase was decanted (this step was repeated three times). The crude peptide was dissolved in deionized H_2_O and lyophilized.

#### 2.3.4 Peptide purification

For purification, the peptides were re-dissolved in H_2_O. To peptides WAW7 and WAW8 a 10-fold excess of dithiothreitol was added, and the mixtures were incubated in an ultrasonic bath at 60°C for 30 min. Each peptide was purified by RP-HPLC with UV-Vis detection (SHIMADZU), using a semi-preparative Luna C8(2) column (250 × 21.2 mm, 5 μm, 100 Å) (Phenomenex). Purification was carried out using a gradient of 5%–50% B in A over 180 min (mobile phase A—H_2_O with 0.1% TFA; mobile phase B—80% acetonitrile in H_2_O with 0.08% TFA). The purity of the peptides was checked by RP-UHPLC with PDA and ELSD-LT detectors (SHIMADZU) using a Kromasil C8 analytical column Kinetex C8 (100 × 2.1 mm, 2.6 μm, 100 Å) and a linear gradient of 5%–100% B in A over 15 min, with detection using an ESI-IT-TOF mass spectrometer (SHIMADZU).

#### 2.3.5 Peptide oxidation assay

The peptide oxidation assay was performed as described previously (Vivian et al., 2009; Kurth et al., 2014) with slight modification. Briefly, peptide substrates (S1—CSGQGNNNCK; S2—RACSGIENCK) with DOTA (1,4,7,10-tetraazacycclododecane-1,4,7,10-tetraacetic acid) coupled at the N-terminus, and a methylcoumarin amide coupled to the Ɛ-amino group of the C-terminal lysine, were custom synthesized by Eurogentec. The lyophilized peptides were resuspended in 100 mM imidazole (pH 6.0) to a concentration of 2 mM. Freshly prepared 100 mM europium trifluoromethanesulfonate (Eu^3+^) solution was added to each peptide at a final concentration of 4 mM and the mixtures incubated for 5 min at room temperature to permit Eu^3+^ chelation. Eu^3+^ was complexed with DOTA in order to facilitate observation of the oxidation rate of cysteine residues in the substrate peptide, leading to the formation of a disulfide bond. This process brings the methylcoumarin antenna into close contact with the DOTA-Eu^3+^ fluorophore, resulting in an increase in Eu^3+^ fluorescence. Thus, fluorescence could be used to monitor the capacity of Hp0231 or Hp0231_APHA_ to catalyze disulfide bond formation.

Assays were conducted using an Infinite^®^ M Nano + plate reader (Tecan) with an excitation wavelength of 340 nm and emission wavelength 615 nm. There was a 150 µs delay before reading and time-resolved fluorescence was measured over a period of 100 µs. The assay was performed in white 384-well plates (Perkin Elmer, OptiPlate-384). Each well contained 50 μL, which included the reaction buffer consisting of 50 mM MES, 50 mM NaCl, 2 mM EDTA (pH 5.5), 160 nM protein (either Hp0231 or Hp0231_APHA_), 1.6 mM GSSG (oxidized glutathione) and 8 µM substrate peptide which was added last to initiate the reaction. Three control reactions were included in each experiment: 1) reaction buffer, GSSG and a protein; 2) reaction buffer and substrate peptide; 3) reaction buffer, substrate peptide and GSSG. Measurements were made for three replicate samples and mean values are presented, with the standard error of the mean indicated by error bars.

#### 2.3.6 Peptide inhibitory activity assay

The peptide inhibitory activity assay was similar to the peptide oxidation assay, only differing by the addition of potential inhibitory peptides (dissolved in 2.5% DMSO): WAW6 (final concentration of 1 mM), WAW7 (final concentration of 1 mM), or WAW8 (final concentration of 0.75 mM). The reaction mixtures consisted of reaction buffer, GSSG, Hp0231 and a substrate peptide. Five control reactions were included in each experiment: 1) reaction buffer, GSSG, Hp0231 and a substrate peptide; 2) reaction buffer, GSSG, Hp0231 and an inhibitory peptide; 3) reaction buffer, GSSG and a substrate peptide; 4) reaction buffer, GSSG, an inhibitory peptide and a substrate peptide; and 5) reaction buffer, an inhibitory peptide and a substrate peptide. The final volume of each reaction mixture was 50 µL. Three independent measurements (technical replicates) were made for each of three replicate samples (biological replicates) and mean values are presented, with error bars indicating the standard error of the mean for the biological replicates.

#### 2.3.7 Assessment of interactions between inhibitory peptides and Hp0231 or Hp0231_APHA_ using microscale thermophoresis

The purified Hp0231 and Hp0231_APHA_ proteins were suspended in HEPES buffer (20 mM HEPES, 150 mM NaCl, 0.05% (*v*/*v*) TWEEN 20, pH 7.4). Protein concentrations were determined by absorption measurements at 280 nm, using the extinction coefficients ε_280_ = 16,055 M^−1^ × cm^−1^ for Hp0231 and ε_280_ = 15,930 M^−1^ × cm^−1^ for Hp0231_APHA_, calculated using Quest Calculate™ Protein Concentration Calculator (AAT BioQuest, https://www.aatbio.com/tools/calculate-protein-concentration). After adjusting their concentrations to 200 nM, Hp0231 and Hp0231_APHA_ were labeled using a Monolith His-Tag Labeling Kit RED-tris-NTA (NanoTemper Technologies) according to the manufacturer’s instructions. Due to their low solubility in water, the inhibitory peptides WAW6, WAW7 and WAW8 were suspended in PBS buffer with DMSO (137 mM NaCl; 2.7 mM KCl; 10 mM Na_2_HPO_4_; 1.8 mM K_2_HPO_4_; 5 mM MnCl_2_; 0.05% (*v*/*v*) TWEEN 20; pH 7.4% and 5% (*v*/*v*) DMSO), to a final concentration of 1 mM. To assess binding interactions between the Hp0231/Hp0231_APHA_ proteins and the inhibitory peptides, MST measurements were performed using a NanoTemper ^®^ Monolith NT.115 instrument (NanoTemper Technologies). Samples were prepared according to the manufacturer’s instructions and loaded in Premium coated capillaries (NanoTemper Technologies). Measurements were performed using MO.Control v1.6.1 (NanoTemper Technologies) software. Medium and high MST power were applied at 25°C. The results were analyzed using MO.Affinity Analysis v2.3 (NanoTemper Technologies) and Origin 2019 (OriginLab Corporation). At least three independent measurements were performed for each sample.

## 3 Results

### 3.1 Designing Hp0231 peptide-based inhibitors, coarse-grained force field analysis of inhibitory peptide binding and conformational changes in the C-terminal catalytic domain of Hp0231

**FIGURE 1:**
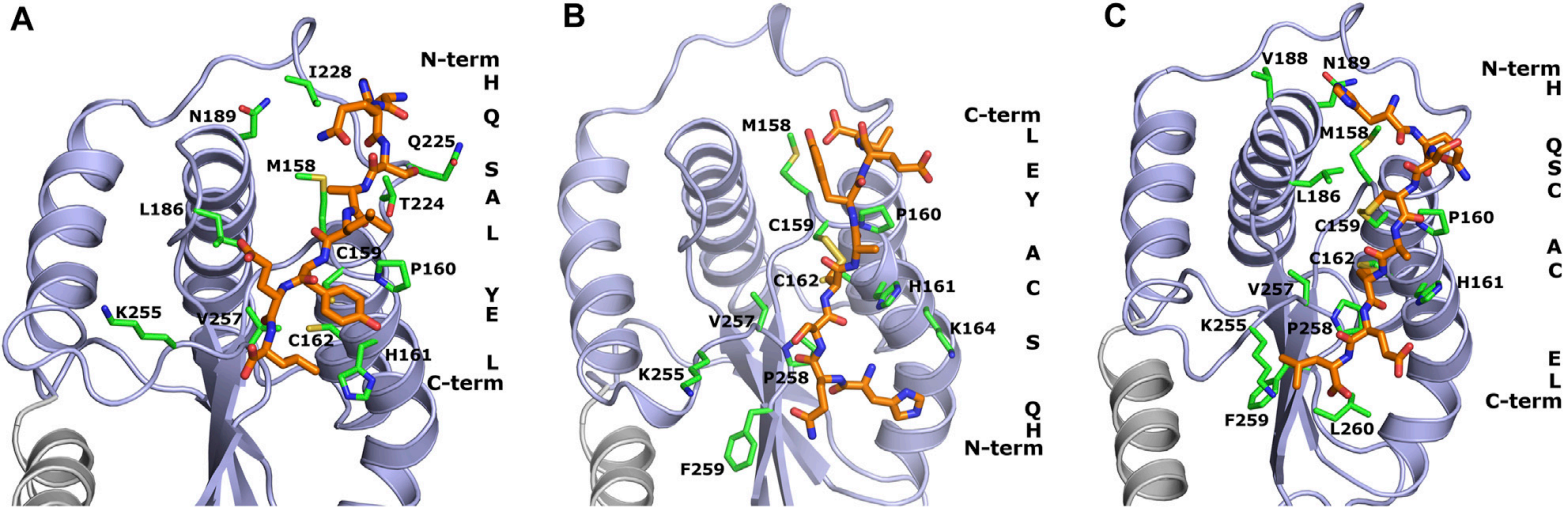
Theoretical models of inhibitory peptides complexed with protein Hp0231 predicted using the UNRES force field. The C-terminal catalytic domain of Hp0231 is colored light blue, while the linker helix is colored gray. The dimerization domain is not shown. Binding conformation of peptides WAW6, WAW7 and WAW8 is presented in panels **(A–C)**, respectively. These inhibitory peptides are colored orange. The inhibitory peptides are colored orange and labeled with a one-letter abbreviation on the right side of each panel. Protein residues located near the peptides are colored green and labeled with their one-letter abbreviations.

The design of peptide-based inhibitors of *H. pylori* oxidoreductase Hp0231 was based on a detailed analysis of amino acid residues located on the protein’s surface close to the catalytic site. The designed sequences (WAW6—Ac-HQSALYEL-NH_2_, WAW7—Ac-HQSCAYEL-NH_2_, WAW8—Ac-HQSCACEL-NH_2_) differ in the number of cysteine residues capable of forming disulfide bridges with the Hp0231 catalytic domain. WAW6 was designed so that it should bind non-covalently in the area of the oxidation site. The lowest energy conformations of WAW6, WAW7 and WAW8 in complex with Hp0231, predicted by the UNRES force field, are presented in Figure 1.

The coarse-grained simulations confirmed that all designed peptides fit to the active site of Hp0231 and moreover are stable during these simulations. Two interaction modes were observed which differ by the location of the peptide backbone (from the N-terminus to the C-terminus). WAW6 and WAW8 bind in a similar way, but simulations demonstrated that the backbone of WAW7 is rotated by 180° in comparison to the other two. A closer examination for non-covalent interactions revealed that residue H1 of the WAW6 peptide is located in a nest created by T224, N225 and I228 of the Hp0231 protein. The glutamine Q2 of WAW6 was placed in the vicinity of residues N189 and M158 of Hp0231. Comparison of the interaction energy (Berka et al., 2010) between glutamine and methionine with that between glutamine and asparagine, suggests that the interaction of the latter residue pair is more important for strong binding of this peptide. One of the most important residues of WAW6 is Y6 (tyrosine), since it is located adjacent to two cysteines in Hp0231, and blocks access to them. In addition, this residue Y6 interacts with P160 (proline) and H161 (histidine). One of the disadvantages of the coarse-grained force field is that reconstruction of the all-atom model is required after the simulation, which means that some information on the sidechain conformation is lost. This drawback is visible for residues T224 (threonine) and K255 (lysine). The threonine is expected to interact with S3 (serine) of inhibitory peptide WAW6 via hydrogen bonding, but the methyl group is directed towards the oxygen atom of this serine residue. However, the most important example of this problematic loss of information during conversion can be seen in the case of K255. The glutamic acid (E7) near the C-terminus of the peptide is oriented towards this residue, but K255 is directed away. In this case, the most probable rotamer of the lysine residue was selected during conversion from a coarse-grained to an all-atom model. The probability of the rotamer being directed towards E7 and the C-terminus of WAW6 was only 4% (Lovell et al., 2000).

Coarse-grained simulations for peptide WAW8 predicted a similar binding conformation to WAW6. However, in this case the peptide is covalently bound to the protein. The most interesting binding mode is represented by peptide WAW7. This is bound covalently to Hp0231 via one cysteine residue. C159 of Hp0231 is located near the protein’s surface, while C162 is buried inside it. In the UNRES simulation, only C159 is able to form a bond with the peptide without large changes to the structure of the catalytic site. Comparison of the binding conformation of WAW7 with that of the other two peptides revealed a 180° rotation. The C-terminus and residue E7 of WAW7 are oriented toward the solvent and H1 forms a cation-π interaction with K164.

### 3.2 Determining the inhibitory activity of peptides towards Hp0231

A two-step chromatography process was utilized to purify His-tagged recombinant Hp0231 and its inactive form Hp0231_APHA_, in which the cysteine residues were substituted with alanines to inactivate the oxidoreductase catalytic motif. The purified proteins were used for further analyses.

Initially we performed an *in vitro* peptide oxidation assay to evaluate the dithiol oxidase activity of Hp0231 towards substrate peptides. Two peptide substrates were employed in this assay: S1, derived from BabA (the blood-group antigen-binding adhesin), an adhesion molecule found in *H. pylori*, and S2, which was artificially designed. Both substrate peptides were synthesized with europium at the N-terminus and methylcoumarin attached to a lysine residue at the C-terminus. This labeling facilitated measurement of increased europium fluorescence occurring upon oxidation of the cysteine residues within the peptide, i.e., disulfide bond formation (Vivian et al., 2009). Furthermore, to enable Hp0231 reoxidation, we conducted the assay in the presence of oxidized glutathione (GSSG).

**FIGURE 2:**
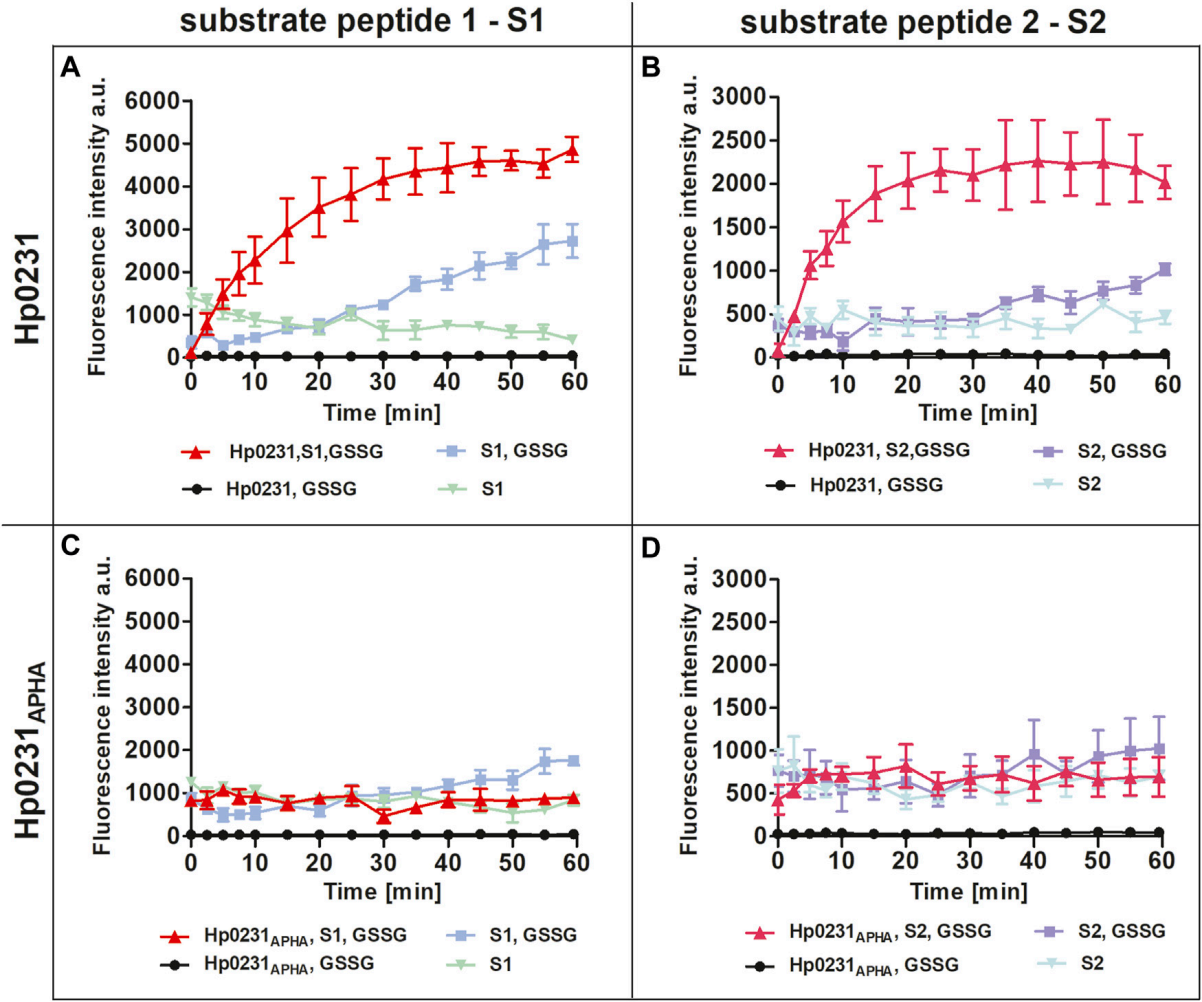
Peptide oxidation assay. Disulfide oxidation activity was measured by an increase in Eu^3+^ fluorescence upon oxidation of peptide substrates S1 **(A, C)** and S2 **(B, D)** in the presence of Hp0231 **(A, B)** or Hp0231_APHA_ **(C, D)**. Appropriate control reactions were also monitored.

Our results unequivocally demonstrated that Hp0231 catalyzes peptide thiol oxidation, as evidenced by a rapid increase in the fluorescent signal over time (Figures 2A, B). As a negative control, we utilized Hp0231_APHA_, which lacks oxidative activity. Hp0231_APHA_ did not catalyze peptide thiol oxidation, so there was no increase in the fluorescence signal over time (Figures 2C, D). Notably, we observed an increase in fluorescence in reactions containing only the peptide substrates and GSSG, albeit at a slower rate than when Hp0231 was present. These results indicated that Hp0231 facilitates dithiol oxidation of both peptide substrates, thus providing an insight into its enzymatic function.

**FIGURE 3:**
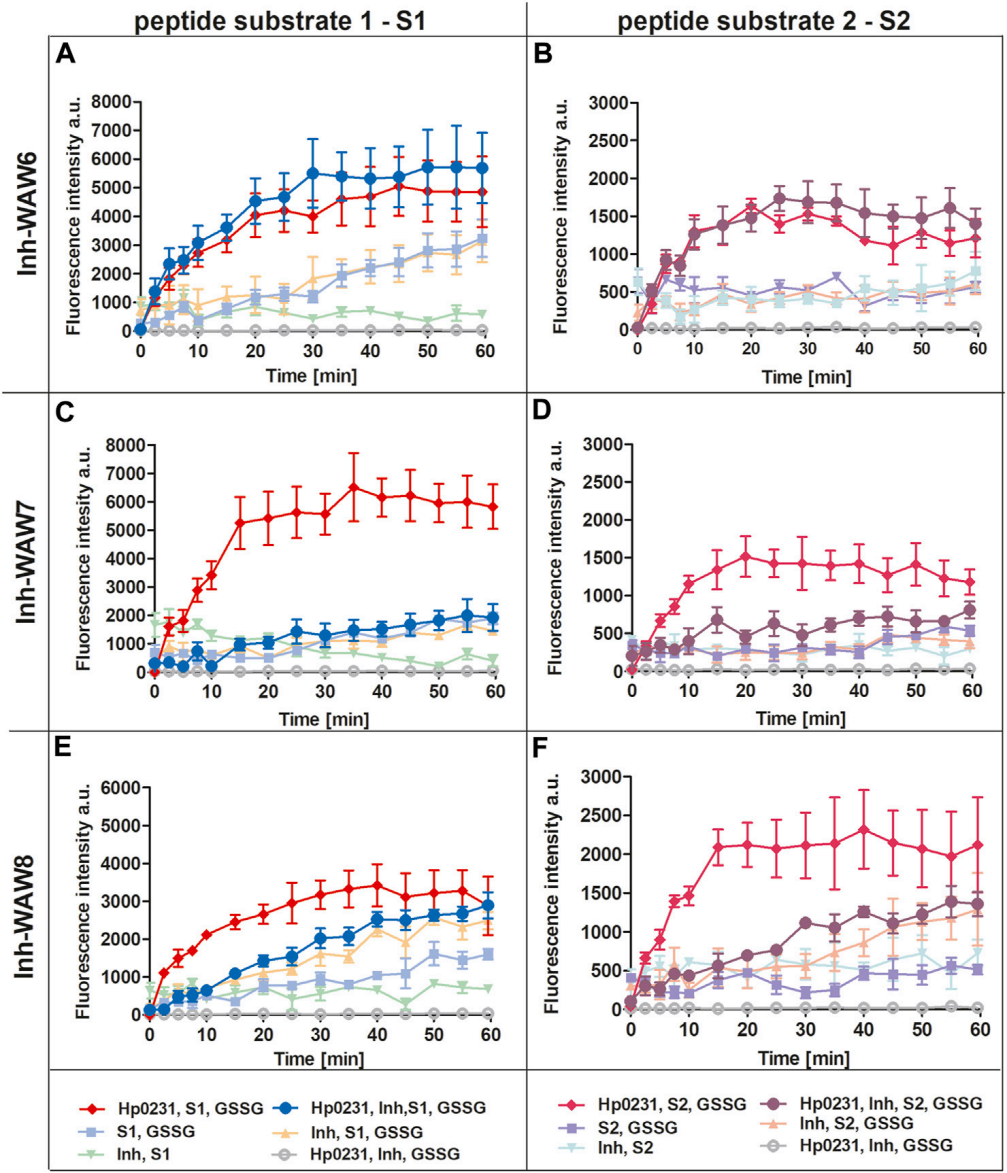
Peptides WAW7 and WAW8 show inhibitory activity against Hp0231 in the peptide oxidation assay. Disulfide oxidation activity was measured by an increase in Eu^3+^ fluorescence upon oxidation of peptide substrates S1 **(A, C, E)** and S2 **(B, D, F)** in the presence of Hp0231. The inhibitory effects of WAW6, WAW7 and WAW8 were evaluated in reaction mixtures containing these potential inhibitory peptides plus appropriate control reactions: WAW6 **(A, B)**, WAW7 **(C, D)** and WAW8 **(E, F)**.

To assess the potential of synthetic peptides to inhibit the interaction between Hp0231 and its substrate peptides (S1 and S2), we added them to the peptide oxidation assay (Figure 3). Addition of peptide WAW6 (Ac-HQSALYEL-NH_2_) to the main reaction mixture (containing Hp0231, GSSG and a substrate peptide) did not yield a significant alteration in the fluorescence intensity of europium (Figures 3A, B). This suggested that WAW6 was not an effective inhibitor of Hp0231-catalyzed oxidation of cysteine residues in the substrate peptides. In contrast, the addition of WAW7 (Ac‐HQSCAYEL‐NH_2_) resulted in inhibition of the fluorescence intensity increase caused by oxidation of the peptide substrates, with Eu^3+^ fluorescence comparable to some of the control reactions (Figures 3C, D). These results indicated that WAW7 was able to block the interaction between Hp0231 and its substrates.

We also evaluated the inhibitory potential of a third peptide WAW8 (Ac‐HQSCAYEL‐NH_2_), which contains two cysteine residues within its sequence. The increase in fluorescence intensity was not inhibited by the addition of WAW8 to the main reaction mixture (containing Hp0231, GSSG and a substrate peptide) (Figures 3E, F). However, the reaction rate was significantly reduced compared to that of the reaction without WAW8. We also noted a similar trend in fluorescence intensity in control reactions containing WAW8, GSSG and S1/S2. These observations suggested that the presence of the second cysteine residue in WAW8 may induce a disulfide exchange between this inhibitory peptide and the substrate peptide, resulting in oxidation of the latter, when an oxidative agent such as GSSG is present.

### 3.3 Characterizing the interaction between peptide-based inhibitors and Hp0231 using microscale thermophoresis (MST)

**FIGURE 4:**
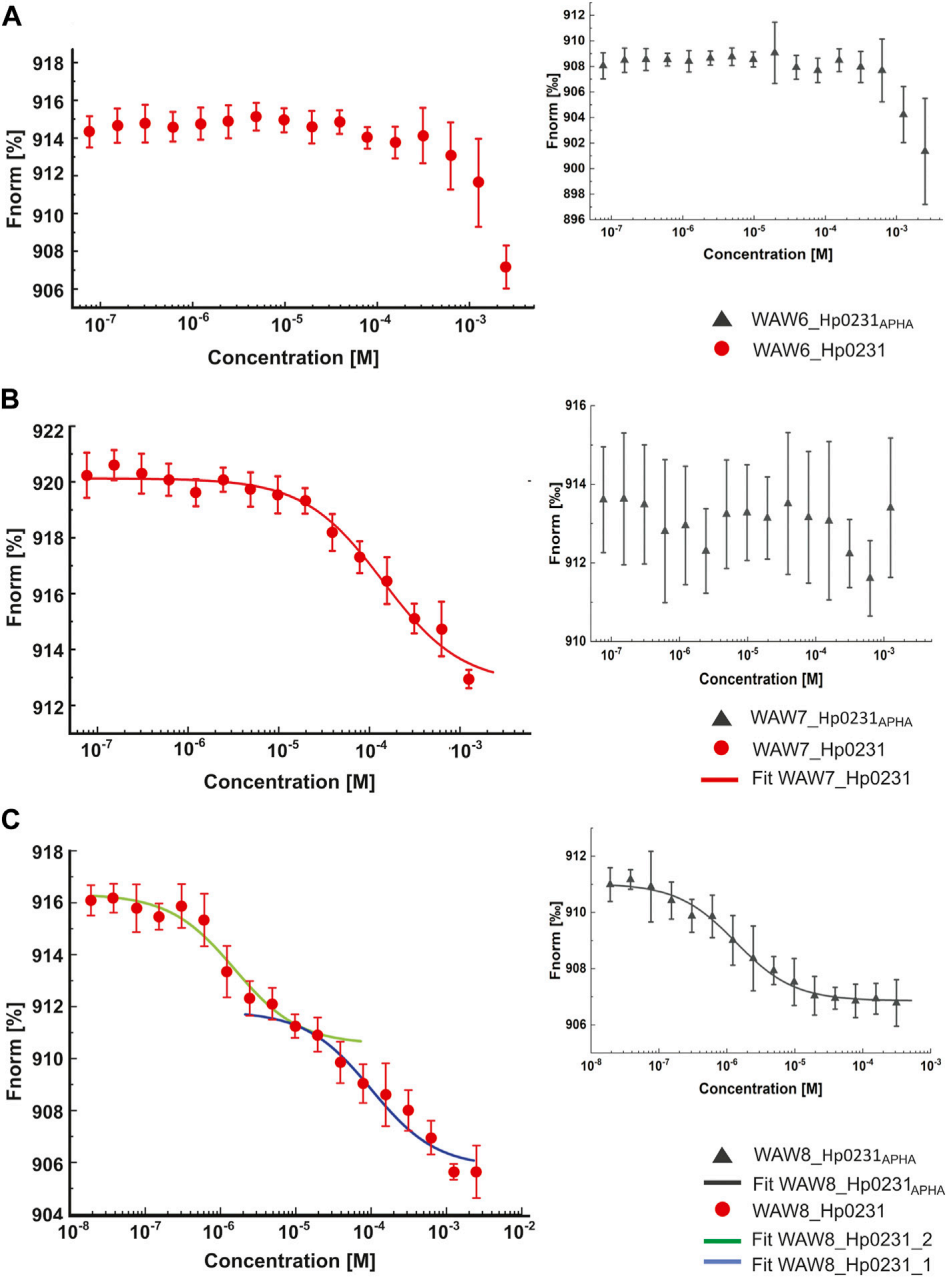
Binding affinity between inhibitory peptides and Hp0231 and Hp0231_APHA_ determined by MST. Titration series of WAW6 **(A)**, WAW7 **(B)**, and WAW8 **(C)** to Hp0231 (red) and Hp0231_APHA_ (gray). MST fitting was done using NanoTemper data analysis software. WAW6 did not bind to either protein. WAW7 interacted with Hp0231 with low affinity and WAW8 showed two binding affinities (low and high).

The MST technique was employed to investigate the interactions between peptides WAW6, WAW7 and WAW8, and Hp0231 protein and its inactive form Hp0231_APHA_. The MST results are presented in Figure 4 and the equilibrium dissociation constants *K*_D_, estimated in pseudo-titration mode, are summarized in Table 2.

**TABLE 2:** Equilibrium dissociation constants (*K*_*D*_) measured by MST.

Titrant/Protein	*K*_*D*_, µM
WAW6/Hp0231	NB
WAW6/Hp0231_APHA_	NB
WAW7/Hp0231	112 ± 21
WAW7/Hp0231_APHA_	NB
WAW8/Hp0231	1.6 ± 0.5, 100 ± 29
WAW8/Hp0231_APHA_	1.3 ± 0.2

The raw MST, data were fitted to obtain *K*_*D*_, using the Nanotemper built-in software. The *K*_*D*_, values are the means from three independent experiments ± S. D. NB, not binding.

As expected, WAW6 demonstrated no binding to Hp0231 or Hp0231_APHA_ (Figure 4A). In contrast, WAW7 interacted with Hp0231 (*K*_D_ = 112 ± 21 μM) (Figure 4B). Notably, the inactive form of the protein showed no interaction with WAW7, suggesting that binding occurs through the catalytic motif of Hp0231. Furthermore, we identified two binding sites in WAW8, with high and low affinity towards Hp0231 (*K*_D1_ = 1.6 ± 0.5 μM and *K*_D2_ = 100 ± 29 μM, respectively) (Figure 4C). Remarkably, only the high-affinity binding site of WAW8 was retained in Hp0231_APHA_ with *K*_D_ = 1.3 ± 0.2 μM (Figure 4C). This indicated that WAW8 can bind Hp0231 at a site outside its catalytic motif. These results provided valuable insights into the interactions between Hp0231 and inhibitory peptides, which can potentially be utilized in the development of novel therapeutic strategies against *H. pylori* infection.

## 4 Discussion

Several human pathogens, including *H. pylori*, *Campylobacter jejuni*, *Salmonella enterica*, *Neisseria meningitidis*, *Vibrio cholerae* and uropathogenic *E. coli* (UPEC), rely on Dsb redox systems to facilitate the deployment of virulence proteins such as adhesion and motility factors, secretion systems and toxins, which are essential for establishing and sustaining infections (Pandeya et al., 2020). Typically, the constituents of Dsb systems are considered non-essential for bacterial viability. Therefore, inhibitors targeting specific components of these systems are unlikely to impede bacterial growth but could attenuate virulence. Dsb oxidoreductases are widespread among bacteria, however there are differences in the Dsb-based oxidation pathway.

Recent research in the field of disulfide bond formation in Gram-negative bacteria has been focused on the identification of inhibitors that can block interactions between DsbAs and DsbB or impede the electron flow from the periplasm to the cytoplasm, which is critical for DsbA regeneration. The low sequence identity of these enzymes with human thiol oxidoreductases suggests potential therapeutic applications of Dsb oxidation pathway inhibitors (Carvalho et al., 2006).

Previously, Duprez et al. (2015) found that short peptides derived from the DsbB P2 loop were able to inhibit the function of *E. coli* DsbA. The nonapeptide (PSPFATCDF) was observed to bind to DsbA with a K_D_ of 4 µM. Modification of the peptide sequence led to a lower K_D_ of approximately 2.9 µM. In the study of Halili et al. (2015), synthetic analogs of ubiquinone (cofactor of DsbB) were used to inhibit disulfide bond formation catalyzed by the *E. coli* DsbA-DsbB system. The mechanism involved covalent modification of single free cysteine residues in both proteins, and the IC_50_ (the half-maximal inhibitory concentration) was around 1 µM. Another approach that has been used to generate inhibitors of DsbA is computer modeling-assisted design of peptidomimetic molecules that bind non-covalently in the hydrophobic groove of this protein disulfide oxidoreductase. However, these molecules proved to be less effective than peptide inhibitors due to their weak and reversible binding (Duprez et al., 2015). Adams et al. (2015) and Totsika et al. (2018) used fragment-based screening to search for inhibitors of *E. coli* DsbA, its homologs in UPEC strains (DsbL) and *Salmonella typhimurium* SrgA. The molecules identified in this way were derivatives of phenylthiazole, phenylthiophene and phenoxy phenyl compounds, and they were shown to affect motility, but not the viability of the studied bacteria. Duncan et al. (2019) employed a fragment-based approach to search for inhibitors of *E. coli* DsbA. They synthesized a series of compounds based on a scaffold that was identified by *in silico* screening, namely, 2-(6-bromobenzofuran-3-yl)acetic acid. The two compounds exhibiting the highest binding affinity (K_D_ values of approx. 350 μM) both bound to the same hydrophobic groove in the structure of DsbA (Duncan et al., 2019). Recently, Wang et al. (2022) also utilized a fragment-based screening approach to identify a small molecule based on the benzimidazole scaffold, which binds in the hydrophobic groove of oxidized DsbA of *V. cholerae* with a K_D_ of 446 µM.

Hp0231 was the first dimeric Dsb described to function in the oxidative pathway of *H. pylori* (Yoon et al., 2011; Roszczenko et al., 2012). Its periplasmic localization makes Hp0231 more accessible for inhibition by small molecules than cytoplasmic targets. The results of our study demonstrate that Hp0231 is capable of catalyzing the dithiol-disulfide oxidation of peptide substrates, providing important insights into the enzymatic function of this protein. Hp0231 catalyzes the formation of disulfide bonds within peptides containing two cysteine residues, regardless of whether the peptide is derived from a natural substrate (such as *H. pylori* adhesin BabA) or is artificial.

Our findings also revealed the inhibitory potential of synthetic peptides against Hp0231-mediated peptide oxidation. The inhibitory peptide was designed following a detailed analysis by computational modeling of the structure of Hp0231 in the vicinity of the cysteine residues.

The peptide WAW6 was found to be an ineffective inhibitor of Hp0231. Indeed, from the obtained results it appears that WAW6 can potentially function as a weak activator of this enzyme (Figures 4A, B). The UNRES forcefield predicted that the conformation is stable (Figure 1), which contradicts the MST measurements (see Table 1) and fluorescence results.

The strongest inhibitory activity was observed in the case of WAW7. This peptide possesses only one cysteine residue, which formed a disulfide bond with C159 of Hp0231. Since the primary function of the protein is oxidase activity, the peptide must have two cysteine residues to make it a substrate for Hp0231. Therefore, after binding, this peptide could not detach from the protein since there is no other cysteine residue in its sequence. As a consequence, WAW7 was a strong inhibitor of Hp0231.

The third peptide examined in this study, WAW8, contains two cysteine residues, but it also displayed inhibitory activity, although much weaker than that of WAW7. Closer examination of the results of molecular modeling and the fluorescence plots (Figures 1, 4) demonstrated that after 1 hour, the curves (with and without inhibitor) are quite similar. An explanation of these results lies in the protein function itself. As both the inhibitory peptide and the substrate peptide contain two cysteine residues, Hp0231 catalyzed disulfide bond formation in both of them. The binding affinity of Hp0231 to the peptide inhibitor appears to be higher than its affinity towards the peptide substrates, but the oxidation process occurred in both peptides over time. When most of the WAW8 cysteine residues had formed disulfide bonds, there was no inhibitor left in the reaction to stop the process. As a consequence, the fluorescence started to increase.

Further studies are required to elucidate the precise mechanism of Hp0231-mediated peptide oxidation and to explore the potential therapeutic applications of *in silico*-designed inhibitory peptides affecting this process.

## Data Availability

The original contributions presented in the study are included in the article/supplementary material, further inquiries can be directed to the corresponding author.
